# Rethinking Clinical Visibility in Autistic Females: Autistic Camouflaging and Menstrual Cycle Variability

**DOI:** 10.3390/ijerph23081006

**Published:** 2026-07-31

**Authors:** Gabrielle A. Grant, Alana Gall

**Affiliations:** National Centre for Naturopathic Medicine, Southern Cross University, Military Road, Lismore, NSW 2480, Australia; gabrielle.grant@scu.edu.au

**Keywords:** autism spectrum disorder, women’s health, menstrual cycle, autistic camouflaging, autism in women, neurodivergence, diagnostic inequity, mental health, late autism diagnosis

## Abstract

**Highlights:**

**Public health relevance—How does this work relate to a public health issue?**

- Autistic females continue to experience delayed diagnosis, misdiagnosis, and inequitable access to appropriate care globally.
- Diagnostic inequities in autism reflect larger and continuing challenges in women’s health, where female presentations and experiences have been historically under-recognised in research and healthcare.

**Public health significance—Why is this work of significance to public health?**

- Autistic camouflaging has been associated with delayed diagnosis and poorer mental health outcomes, highlighting an important but under-recognised women’s health concern.
- Fluctuations in the menstrual cycle may influence domains that overlap with those required for camouflaging, representing an overlooked area of autism research.

**Public health implications—What are the key implications or messages for practitioners, policy makers and/or researchers in public health?**

- Greater consideration of factors that influence clinical visibility may support more equitable autism recognition, diagnosis, and access to care for autistic females.
- Future research, policy, and clinical practice should be informed by the lived experience of autistic females to improve diagnostic equity and health outcomes.

**Abstract:**

Autism spectrum disorder (hereafter autism) is increasingly recognised as being underdiagnosed in females worldwide, contributing to inequalities in clinical visibility and access to appropriate care. The clinical visibility of autism in females is influenced by diagnostic practices historically developed using predominantly male samples, which may be less sensitive to less overt or more socially camouflaged presentations. Greater levels of autistic camouflaging, including masking, compensations, and assimilation, have been proposed as one factor contributing to differences in the presentation of autism between males and females. However, camouflaging is resource-intensive, associated with significant psychological costs, and varies across contexts. Because camouflaging relies on cognitive–emotional resources, it may also fluctuate under conditions that influence these resources, such as the menstrual cycle. To date, no empirical research has directly investigated this relationship. This opinion article proposes the conceptual hypothesis that menstrual cycle-related fluctuations may influence the cognitive–emotional resources required for autistic camouflaging, with potential implications for clinical visibility, diagnostic recognition, mental health, and equitable access to care. By synthesising indirect evidence from autism, camouflaging, and menstrual health research, this conceptual framework aims to guide future empirical investigation and highlights the urgent need to better understand this overlooked intersection to improve diagnostic equity, clinical recognition, and women’s health outcomes globally.

## 1. Introduction

Disparities in equitable healthcare access and recognition remain a public health concern for women globally. Women have historically been excluded from health and medical research. Despite policy reforms, women remain underrepresented in some areas of clinical and preclinical research, with sex- and gender-specific analyses still inconsistently embedded in study design, analysis, and reporting [1]. For example, research guidelines were updated in the early 1990s to mandate the inclusion of women in clinical trials, but the impacts of prolonged underrepresentation still affect healthcare and diagnostic practices today [2]. While women and girls have been documented as autistic since at least the early 1900s, it was not until the 2010s that increased research into the reasons for and implications of differences in presentations between males and females began [3]. Consequently, diagnostic tools were predominantly developed with male samples [4,5]. These inequities are increasingly recognised in the misdiagnosis, and late diagnosis of autism spectrum disorder (hereafter autism or autistic individual for females [6]. The authors note that identify-first language has been used to refer to autism and autistic people, as this is currently the most preferred means of identification [6]) The authors acknowledge that not all individuals included in research on female autism or menstrual health identify as women, girls, or female. For the purposes of consistency and brevity, the terms “females, women, and/or girls” are used throughout this paper when referring to individuals identified as female within the cited literature and/or individuals who currently experience or have previously experienced menstrual cycles. This term is not intended to exclude gender-diverse individuals [7]. Moreover, autistic females represent a highly heterogenous population with considerable variation in symptom presentation, intellectual functioning, co-occurring conditions, and potentially hormonal experiences [5,8,9]. These individual differences are likely to influence the expression of autistic characteristics and the extent to which camouflaging may contribute to clinical visibility.

An emerging body of research posits a contributor to the underdiagnosis of autistic females may be the greater use of camouflaging behaviours (the autistic community often refer to camouflaging behaviours simply as masking, but for the purposes of this paper, we will be referring to this set of behaviours with the term camouflaging [10]). Camouflaging is a broad term which encompasses behaviours often employed by autistic individuals to blend in with society and avoid social exclusion [11]. The concept of camouflaging includes three broad behaviours that can be conscious or unconscious: the masking of autistic traits, compensatory strategies to account for social difficulties, and assimilation into the social environment [12,13]. Autistic females utilise camouflaging at a significantly higher rate than autistic males [11]. Researchers have suggested that gendered social expectations are a contributing factor to this overutilisation, as females have deeply entrenched social expectations to be more empathetic, socially communicative, emotionally expressive, and socially engaged than males [11]. While camouflaging is used to avoid negative social experiences, it poses a significant risk to the mental health of individuals who utilise this strategy and may contribute to late or missed diagnosis [10,14,15]. Additionally, physiological factors that use similar processes to camouflaging, such as the menstrual cycle, have not yet been explored for their potential contribution to camouflaging fluctuations.

Thus far, no empirical research has investigated if menstrual cycle-related fluctuations impact autistic camouflaging. This opinion article presents a conceptual hypothesis by synthesising existing autism, camouflaging, and menstrual health research and proposes that menstrual cycle-related changes may influence the cognitive–emotional resources required to sustain camouflaging. It is further hypothesised that these fluctuations may affect the clinical visibility of autism, diagnostic recognition, and associated mental health outcomes. This opinion article does not seek to establish a causal relationship but integrates indirect evidence from related fields to provide a theoretical framework intended to inform and stimulate future empirical research. Both authors are late-diagnosed autistic females with lived experience with the inequities of our healthcare system. We value the importance of lived experience in all facets of research.

## 2. Diagnostic Inequity for Females

Historically, autism was conceptualised as a male disorder and was more frequently diagnosed in males than females. Recent studies estimate males are three to four times more likely to receive a clinical diagnosis [16]. Moreover, females often receive an autism diagnosis significantly later in life than males, if ever, and are more likely to be misdiagnosed prior to an accurate diagnosis [4]. However, the actual prevalence of autism in females is currently unknown. Suggested contributing factors to this disparity are the male-normative biases embedded within current diagnostic tools and the enduring andronormative understanding of autism [4]. Indeed, gold-standard autism diagnostic tools continue to be insensitive to female-specific presentations of autism [4]. These tools were developed with predominantly male samples and prioritise the recognition of externalised, observable behaviours, and stereotypical, restricted interests like dates in history, mechanics, or physics [4,5]. Consequently, the more subtle female presentations of autism may be missed with existing diagnostic measures [5]. Female presentations often include higher social motivation, internalisation of difficulties, higher levels of empathy, and restricted interests, which may align with socially acceptable gender norms, such as intense interests in psychology, fashion, or animals [4,5]. The male-centric focus of these tools and understanding of autism overlooks the lived experience of autistic females, potentially contributing to delayed support and significant distress.

Late diagnosis of autism in females has consequences beyond delayed access to care, including significant impacts on mental health, social connection, and self-perception. This delay has been associated with increased risk of co-occurring mental health conditions, with the most highly reported being depression, anxiety, suicidality, self-harm, substance misuse, and eating disorders [17,18,19]. Delayed diagnosis alongside these mental health struggles can negatively affect identity and self-perception, contributing to negative self-image, identity confusion, and chronic self-blame [19]. The implications of delayed diagnosis can also have broader social consequences, including social isolation, exposure to risky situations, and increased risk of sexual abuse [17]. Delayed diagnosis is also often accompanied by misdiagnosis, which may contribute to inappropriate psychiatric treatment and further delays in accessing appropriate interventions and support [17]. Collectively, these outcomes highlight the harmful and disproportionate consequences that autistic females may experience when diagnosis is delayed. They also highlight the need for more appropriate diagnostic tools and clinical practices to reduce inequitable access to care. Diagnostic reform is critical for the reduction in preventable negative mental health outcomes and harmful social experiences associated with delayed or missed diagnosis. One factor suggested as a contributor to missed or delayed diagnosis is autistic camouflaging, which has become a recent focus in autism research [12].

## 3. Camouflaging as a Dynamic and Resource-Dependent Process

Autistic camouflaging has increasingly been investigated as a factor potentially contributing to the underdiagnosis, late diagnosis, and missed diagnosis of autism in females. By reducing visibility, camouflaging may make autistic traits less likely to be recognised during assessment [20]. It may also occur more frequently and intensify over time when autism remains undiagnosed [11]. A greater understanding of the prevalence and complexities of camouflaging in autistic females could potentially support more accurate and timely diagnosis. Camouflaging is described as an adaptive process used by autistic individuals to navigate social environments and meet perceived social expectations [11]. This definition implies variability, as camouflaging behaviours must be adjusted according to context. Consistent with this description, emerging evidence suggests that camouflaging is situational, with higher levels reported in social, unfamiliar, or high-stakes settings, and lower levels in private or safe environments [12,20].

Importantly, camouflaging is not only context-dependent but can be resource-dependent. It may require sustained cognitive effort, attentional control, emotional regulation, monitoring of social cues, suppression of autistic traits, and management of sensory or interpersonal stressors [10,14]. These demands suggest that camouflaging is a taxing process that may fluctuate according to an individual’s available cognitive, emotional, sensory, and physiological resources. Camouflaging may also vary across development and the lifespan, as social expectations, environments, identity formation, coping strategies, and access to diagnosis or support change over time [11]. In the absence of diagnosis, camouflaging may become more frequent or more intense, particularly when individuals lack appropriate recognition, accommodations, or support [11].

Collectively, the dynamic and resource-dependent nature of camouflaging suggests that it may be susceptible to fluctuations in internal and external demands. A plausible, yet unexplored, source of such variability is the menstrual cycle. The menstrual cycle is a naturally occurring physiological process associated with fluctuations in cognitive–emotional domains, the same domains that are vital to the achievement of autistic camouflaging, including attention, emotional regulation, fatigue, and cognitive flexibility [10,14,21,22]. However, the menstrual cycle is proposed as only one potential contributor to within-person fluctuations in camouflaging. Other factors which may influence camouflaging capacity and should be considered alongside the proposed conceptual framework are fatigue, sensory demands, co-occurring mental health conditions, and contextual stressors [8,10,11,20,22]. Accordingly, this opinion article hypothesises that menstrual cycle variability may represent one potential contributor to the cognitive–emotional resources accessible for camouflaging, thus affecting the clinical visibility of autism.

## 4. Menstrual Cycle as a Plausible Modifier

The menstrual cycle was selected as the focus of this conceptual hypothesis as it represents a biological process associated with naturally occurring variations in several cognitive–emotional domains, the same domains needed for autistic camouflaging. The basis for the proposed relationship is founded on the overlap between these domains, rather than evidence of a direct hormonal influence on camouflaging itself. As such, the proposed framework merges indirect evidence from related fields to generate a testable hypothesis, rather than drawing conclusions from direct empirical evidence of the proposed relationship.

There has been increased recognition of the influence the menstrual cycle may have on mood, emotional regulation, cognition, and fatigue. However, there remains a dearth of research on how these fluctuations may affect the clinical diagnostic visibility of autism. Hormonal variations across the menstrual cycle may influence mood, emotional regulation, attention, and fatigue, with emerging evidence suggesting related changes in the brain in the networks that support these processes [21,22]. Whilst caution should be taken when interpreting the existing literature, it provides a useful foundation for the development of the proposed conceptual framework. For instance, dynamic complexity has been seen to have the highest level of variability during the pre-ovulatory phase of the menstrual cycle and the lowest level of variability in the early follicular phase, suggesting that brain flexibility and adaptability fluctuate depending on the menstrual cycle phase [21]. Although findings regarding objective cognitive performance across the menstrual cycle have been mixed, emerging evidence suggests that hormonal fluctuations may influence the neural systems involved with attention, emotional processing, and cognitive flexibility [21,22]. Consequently, the current evidence indicates that changes may be more evident in underlying neural function and subjective experience rather than measurable cognitive performance.

**Figure 1:**
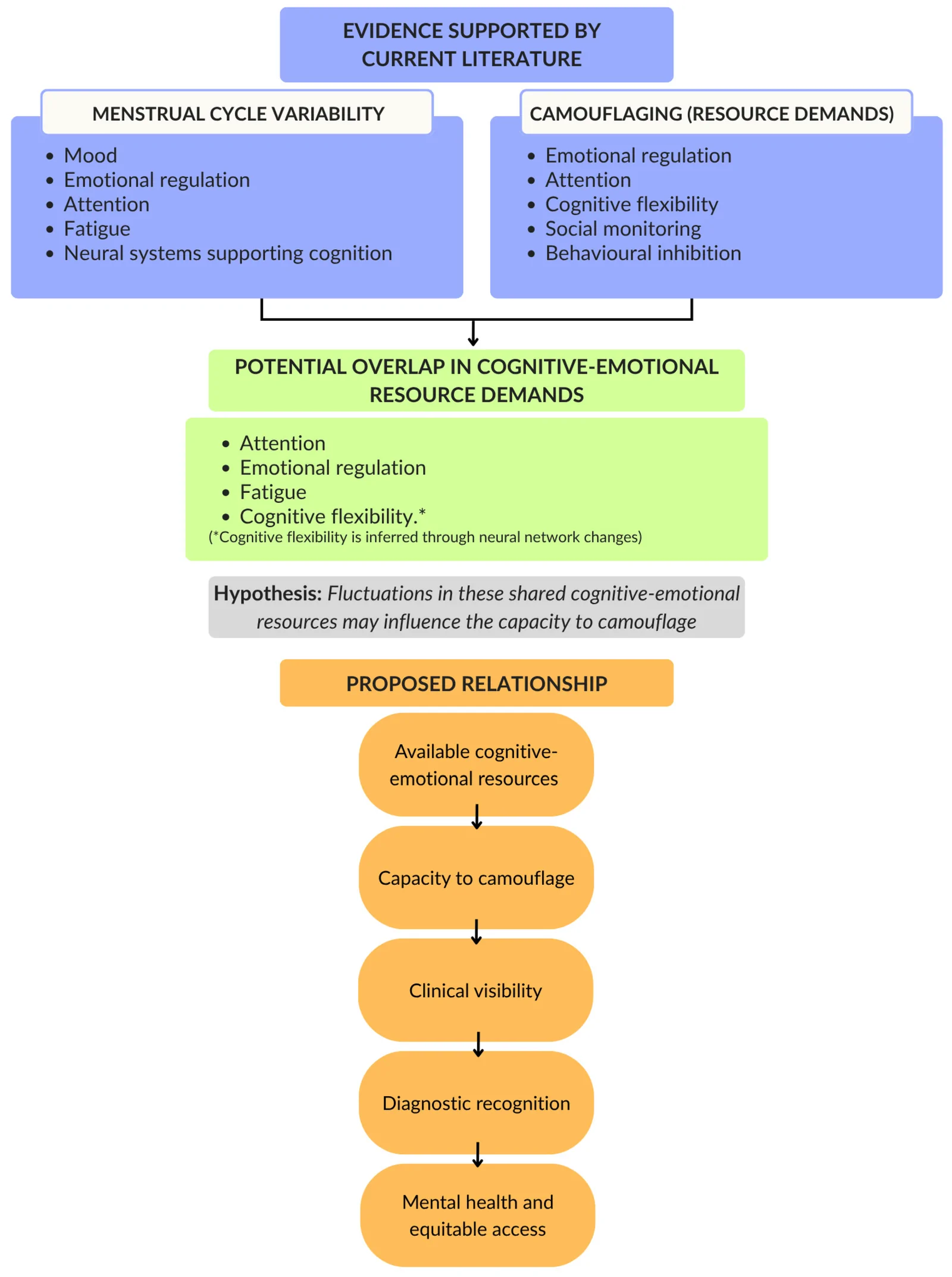
Proposed conceptual framework illustrating the hypothesised relationship between menstrual-cycle-related fluctuations and autistic camouflaging. The blue section summarises evidence supported by the current literature regarding domains potentially influenced by the menstrual cycle [8,21,22] and the cognitive–emotional resource demands of autistic camouflaging [10,12,14,20]. The green section represents the conceptual overlap between these domains. The grey section presents our hypothesis. The orange section illustrates the theoretical pathway proposed in this opinion article, which has not yet been empirically tested.

Similarly, autistic camouflaging involves reliance on sustained attention, emotional regulation, cognitive flexibility, behavioural inhibition, and the capacity to manage fatigue, several of which overlap with domains affected by the menstrual cycle [10,14]. It is this overlap in cognitive–emotional resource demands that forms the basis of the conceptual hypothesis proposed in this opinion article. If these shared resources fluctuate across the menstrual cycle, the capacity to engage in camouflaging may also vary, potentially influencing the clinical visibility of autism. Considering the overlap between resources required for camouflaging and domains affected by the menstrual cycle, it is hypothesised that menstrual cycle variability may influence camouflaging capacity (Figure 1).

However, this proposed relationship has not yet been empirically examined and therefore warrants further investigation. Future research on the proposed hypothesis should consider the heterogeneity of individual hormonal variability, menstrual cycle phases, sensory processing differences, cognitive inflexibility, co-occurring conditions, stress, sleep quality, and social context as potential moderators of the relationship between menstrual cycle variability and the cognitive–emotional resources available for camouflaging. These factors may independently influence camouflaging and should therefore be carefully considered when evaluating the proposed conceptual framework.

## 5. Implications for Mental Health and Equitable Access to Care

Diagnostic inequity experienced by autistic females is a global public health concern with many negative consequences. Females who are diagnosed late have a higher prevalence of chronic mental health conditions such as anxiety and depression [4]. Moreover, late diagnosis prevents individuals from accessing essential support and understanding their neurotype. Not only does this delay access to support, late diagnosis is also often accompanied by misdiagnosis, which may contribute to inappropriate psychiatric care and long-term consequences for wellbeing and social participation [17,19]. Additionally, delayed diagnosis may contribute to higher levels of camouflaging that become more intense with age, particularly for women, with higher levels of camouflaging further masking autistic traits and potentially creating more difficulty for clinicians in diagnostic recognition [11,15]. Camouflaging has repeatedly been argued to have long-term negative consequences for autistic individuals. Chronic camouflaging is associated with high levels of anxiety, depression, burnout, loss of identity, and notably, suicidality [10,14,17]. Given the severe risks associated with late diagnosis and chronic camouflaging, providing equitable access to care is paramount for autistic females.

Policy and practice around diagnosis and care should be amenable to autistic females’ needs. Due to historical misunderstandings and underrepresentation of autistic women in research, there has been a failure to adequately capture autistic female experiences [1,2]. The continuation of research about autistic females without lived experience involvement may perpetuate previous male-centric assumptions and deficit-based frameworks. A vital step towards equitable care includes recognising the value of lived experience voices in the design and development of autism research. The most important reason for the inclusion of lived experience in research is the emphasis it puts on the significance of improving the lives of those most affected by the research [23]. Greater care and responsibility must now be taken by researchers and clinicians to ensure accurate representation of autistic females in research and practice. While the proposed conceptual framework may ultimately have implications for clinical practice if supported by future empirical research, these implications remain preliminary until the hypothesised relationship has been empirically tested.

## 6. Conclusions and Future Directions

Considering the enduring diagnostic inequities experienced by autistic females globally and the negative consequences of these inequities, it is imperative that greater consideration be given to the factors that may influence female clinical visibility. Camouflaging has been argued to be a significant contributor to the underdiagnosis of autism in females, and when combined with delayed diagnosis, it can have severe mental health outcomes [11,13]. Improved understanding of factors that may influence the cognitive–emotional resources required for camouflaging, including menstrual cycle-related variability, may prove important for determining whether these fluctuations contribute to differences in the clinical visibility of autism and to support more equitable access to diagnosis and care. The proposed conceptual framework in this article has been informed by emerging, yet methodologically limited, evidence. Future research should prospectively investigate whether naturally occurring physiological variability contributes to within-person changes in autistic camouflaging and clinical visibility. However, care should be taken when considering whether menstrual cycle variability enhances or complicates clinical assessment and in ensuring this addition does not reinforce gender-based stereotypes. Integrating longitudinal approaches capable of capturing fluctuations over time, together with quantitative measures and lived experiences of autistic individuals, will be essential for assessing the suggested conceptual framework. Failure to consider these influences risks overlooking factors that may contribute to the ongoing diagnostic experience of autistic females. If the proposed framework is supported by future empirical evidence, it may advise future diagnostic guidelines that acknowledge the temporal variability in autistic presentation for multidisciplinary care, the development of clinical guidelines, and policy reform through meaningful collaboration with autistic females, clinicians, researchers and other stakeholders.

## Data Availability

No new data were created or analyzed in this study.
